# Mutual interplay between phytopathogenic powdery mildew fungi and other microorganisms

**DOI:** 10.1111/mpp.12771

**Published:** 2019-02-18

**Authors:** Ralph Panstruga, Hannah Kuhn

**Affiliations:** ^1^ Unit of Plant Molecular Cell Biology, Institute for Biology I RWTH Aachen University Worringerweg 1 Aachen 52056 Germany

**Keywords:** induced systemic resistance, microbiome, microbiota, mycoparasites, powdery mildew

## Abstract

Powdery mildew is a common and widespread plant disease of considerable agronomic relevance. It is caused by obligate biotrophic fungal pathogens which, in most cases, epiphytically colonize aboveground plant tissues. The disease has been typically studied as a binary interaction of the fungal pathogen with its plant hosts, neglecting, for the most part, the mutual interplay with the wealth of other microorganisms residing in the phyllo‐ and/or rhizosphere and roots. However, the establishment of powdery mildew disease can be impacted by the presence/absence of host‐associated microbiota (epi‐ and endophytes) and, conversely, plant colonization by powdery mildew fungi might disturb indigenous microbial community structures. In addition, other (foliar) phytopathogens could interact with powdery mildews, and mycoparasites may affect the outcome of plant–powdery mildew interactions. In this review, we discuss the current knowledge regarding the intricate and multifaceted interplay of powdery mildew fungi, host plants and other microorganisms, and outline current gaps in our knowledge, thereby setting the basis for potential future research directions.

## The Plant–powdery mildew pathosystem

Powdery mildew is a ubiquitous and frequent disease of many angiosperm plant species (Glawe, 2008). It is caused by obligate biotrophic fungi of the order Erysiphales (Braun and Cook, 2012). The interaction between mono‐ and dicotyledonous plant hosts and powdery mildew fungi serves as a paradigm to study various aspects of molecular phytopathology. These include different types of plant defence mechanisms [e.g. basal defence, non‐host resistance, quantitative (multigenic) resistance, resistance (*R*) gene‐mediated immunity, *mildew resistance locus o* (*mlo*)‐based resistance], but also crucial aspects of fungal pathogenesis and the obligate biotrophic lifestyle (Hückelhoven and Panstruga, 2011; Jørgensen, 1994; Kuhn *et al.*, 2016; Micali *et al.*, 2008; Schweizer, 2007). Powdery mildews exclusively thrive on nutrients taken up from the plant host—a lifestyle termed obligate biotrophy. Powdery mildew colonization therefore affects the partitioning of carbohydrates and other nutritive substances within the plant by generating a strong local sink at foliar fungal infection sites (Edwards, 1971; Wright *et al.*, 1995). In addition, powdery mildews are highly effective in suppressing host defence (Panstruga, 2003) and in creating a specialized leaf microenvironment, termed the ‘green island’ (Walters and McRoberts, 2006). Given these exquisite features, it is conceivable that successful powdery mildew colonization will affect pre‐existing foliar and, possibly, even root/rhizosphere‐associated microbial communities.

**Figure 1:**
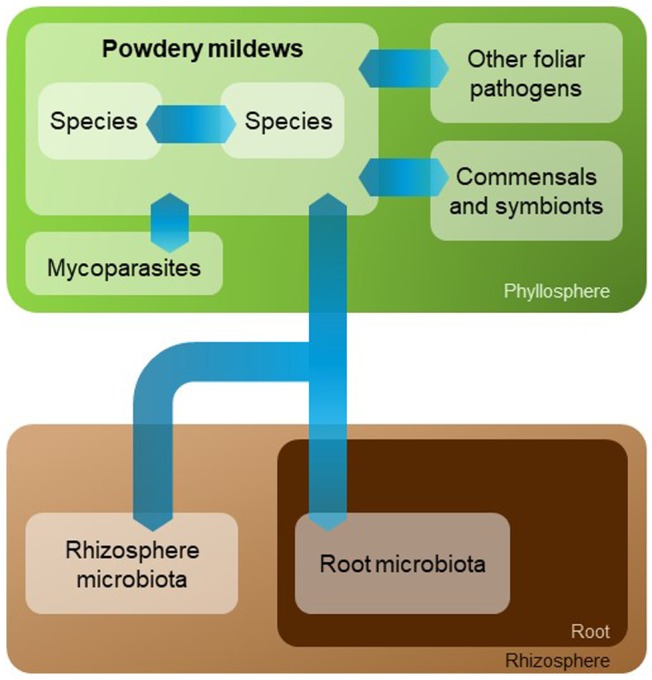
Mutual interplay between phytopathogenic powdery mildew fungi and other plant‐associated microorganisms. The scheme illustrates the various possible types of interaction (blue arrows) between powdery mildew fungi, their different species and other microorganisms, such as phyllosphere and root/rhizosphere microbiota (commensals, pathogens and symbionts), as well as mycoparasites.

Binary interactions between plant hosts and powdery mildew fungi have been studied extensively and are well characterized. For example, the interaction between the model plant *Arabidopsis thaliana* and its adapted powdery mildew pathogen *Golovinomyces orontii* has been analysed thoroughly in the past. Natural genetic variation of the degree of powdery mildew susceptibility between ecotypes has been documented comprehensively (Adam *et al.*, 1999; Göllner *et al.*, 2008), and a broad panel of induced mutants with either enhanced disease resistance or enhanced disease susceptibility is available (Kuhn *et al.*, 2016). Methods for the (semi‐)quantitative evaluation of infection phenotypes are fully established. These comprise the assessment of infection success at early (host cell entry), medium (hyphal expansion) and late (conidiation) stages of fungal pathogenesis (Baum *et al.*, 2011; Seiffert and Schweizer, 2005; Weßling and Panstruga, 2012). However, as in the case of most other investigated plant–microbe encounters, the vast majority of studies conducted on fungal powdery mildew pathogens disregard the potential effect of other microbes (further pathogens and/or the host‐associated bacterial and fungal microbiota) on the outcome of the interaction and the establishment of disease. In addition, it is currently not known whether and how colonization of a plant by powdery mildew affects the composition of the indigenous host bacterial and fungal microbiota in the phyllosphere and, possibly even via systemic effects, in the roots/rhizosphere (Fig. 1).

## Induced systemic resistance

Induced systemic resistance (ISR) describes a physiological state of elevated immune responsiveness (‘defence priming’) which is, for example, triggered by plant growth‐promoting bacteria and fungi residing in the rhizosphere (Pieterse *et al.*, 2014). Local suppression of root immune responses is a common feature of ISR‐eliciting beneficial microbes. ISR triggered by beneficial soil‐borne microbes is often regulated by jasmonic acid/ethylene‐dependent signalling pathways, but beneficial microbes that elicit the salicylic acid (SA)‐dependent pathway, which is classically linked to pathogen‐induced systemic acquired resistance (SAR), also exist (Knoester *et al.*, 1999; Pieterse *et al.*, 1998; Pozo *et al.*, 2008). The transcriptional regulator MYB72, which is expressed in root tissue, is a key local regulator of ISR. *Arabidopsis* *myb72* mutants fail to trigger ISR responses against different foliar pathogens on treatment with the bacterial ISR inducers *Pseudomonas fluorescens* WCS417r or *Pseudomonas putida* WCS358r, indicating that this root‐specific transcription factor is essential for the onset of ISR (van der Ent *et al.*, 2008). More recently, transcriptomic studies have revealed additional genes with a presumptive role in ISR (Mathys *et al.*, 2012).

## Root/rhizo‐ and phyllosphere microbiota

The core root/rhizo‐ and phyllosphere‐associated bacterial microbiota (i.e. the ecological community of commensal, symbiotic and pathogenic microorganisms) of several plant species have been resolved during the last few years using mainly culture‐independent (ribosomal DNA sequencing) methods, in part complemented by the large‐scale cultivation of organ‐specific microbes. Particularly well studied in this respect are the dicotyledonous plant *Arabidopsis* and the monocotyledonous crop barley (*Hordeum vulgare*). Via pyrosequencing of bacterial 16S ribosomal RNA (rRNA) gene amplicons, *Arabidopsis* roots of plants grown under controlled conditions in natural soils were found to be preferentially colonized by Proteobacteria, Bacteroidetes and Actinobacteria, with each bacterial phylum represented by a dominating class or family. These phyla thus define the ‘bacterial core microbiota’ of *Arabidopsis* roots and are significantly enriched in roots relative to the soil and rhizosphere. Both considerable soil‐type and moderate genotype effects were noted with respect to the composition of the root microbiota (Bulgarelli *et al.*, 2012; Lundberg *et al.*, 2012; Schlaeppi *et al.*, 2014). Similar results regarding the composition of the root microbiota of *Arabidopsis* roots were obtained with samples from *Arabidopsis* collected in natural habitats (Bodenhausen *et al.*, 2013) and by the cultivation of root‐associated bacteria (Bai *et al.*, 2015).

The analysis of the bacterial root microbiota of various barley genotypes revealed overlapping and distinctive features relative to *Arabidopsis*. In barley, a marked ‘rhizosphere effect’, i.e. a structural and phylogenetic diversification of this microhabitat from the surrounding soil biota, was discovered. Taxonomic classification of the actual root microbiota revealed a similar enrichment pattern to *Arabidopsis*, but with some distinctive features. The bacterial families Comamonadaceae, Flavobacteriaceae and Rhizobiaceae dominate the barley root‐enriched microbiota. The enrichment of members of the families Pseudomonadaceae, Streptomycetaceae and Thermomonosporacea, seen in the root samples of *Arabidopsis*, was not observed in barley. By contrast, enrichment of members of the Microbacteriaceae family appears to be a distinct feature of the barley root microbiota. Nevertheless, the overall conserved microbiota composition in and at the roots of monocotyledonous barley and dicotyledonous *Arabidopsis* could be indicative of an ancient plant trait (Bulgarelli *et al.*, 2015). It should be noted, however, that the analyses in barley and *Arabidopsis* were performed with different primers and sequencing approaches, and the respective data were not quantitative, limiting the comparability.

Although the focus of the majority of previous work was clearly on the root and bacterial side, systematic studies on fungal (Agler *et al.*, 2016; Wang *et al.*, 2016) and/or phyllosphere (Bodenhausen *et al.*, 2014) microbiota have begun to complete the picture of the plant holobiont. In one of these studies, it was found that the microbial community structure of the *Arabidopsis* phyllosphere is governed by a network of interkingdom interactions, in which a small number of key microbial taxa (‘hubs’) are strongly interconnected and have a severe impact on the community (Agler *et al.*, 2016).

## Local microbial effects on powdery mildew colonization

Some bacteria (or their respective culture filtrates) constrain powdery mildew proliferation, and these are in part being used as so‐called biocontrol agents against this fungal disease. Prominent examples in this respect are members of the genus *Bacillus*, which have been repeatedly described to show great potential as biocontrol agents. An instance of this is *Bacillus* sp. BS061. A culture filtrate of this bacterium was found to effectively reduce the disease incidence of powdery mildew on cucumber and strawberry via the secretion of substances with antifungal capacity (Kim *et al.*, 2013). Similarly, leaf treatment with *Bacillus subtilis* strain E1R‐J reduced the disease index of wheat (*Triticum aestivum*) infected with powdery mildew (*Blumeria graminis* f. sp. *tritici*) by affecting powdery mildew infection structures (Gao *et al.*, 2015). A systematic assessment of 169 bacterial seed endophytes isolated from domesticated cucurbits revealed that six isolates (including one *Bacillus *strain) led to suppression of powdery mildew symptoms when applied in a leaf disc/detached leaf assay (Khalaf and Raizada, 2018). In a comparable study, of 22 tested bacterial strains recovered from the phyllosphere of cucurbit plants or different soils, four *Bacillus* isolates showed potent activity in reducing powdery mildew (*Podosphaera fusca*) infection when applied to detached cucurbit leaves and seedlings, leading to up to 80% reduction of disease symptoms (Romero *et al.*, 2004). Reduced powdery mildew (*B. graminis*) incidence and disease index were also observed in drunken horse grass (*Achnatherum inebrians*) colonized by the seed‐borne endophyte *Epichloë gansuensis*. By contrast, no effect of the closely related endophyte *Neotyphodium* sp. was seen on the extent of powdery mildew infection of tall and meadow fescues (Sabzalian *et al.*, 2012).

In addition to microbes (or culture filtrates) which antagonize powdery mildews directly, possibly via antibiosis, the effects of microbes on the stimulation of plant defence, thereby indirectly combating powdery mildews, have been reported. For example, the bacterial biocontrol agent *Bacillus amyloliquefaciens* LJ02 induces defence responses in cucurbit seedlings that are effective in limiting the extent of powdery mildew (*Sphaerotheca fuliginea*) colonization. Treatment of the seedlings with the bacterium increased the levels of the defence‐related phytohormone SA, stimulated the expression of *pathogenesis‐related gene 1* *(PR‐1*) and resulted in higher activities of the defence‐related enzymes superoxide dismutase, peroxidase, polyphenol oxidase and phenylalanine ammonia lyase (Li *et al.*, 2015). Similarly, pretreatment of pea (*Pisum sativum*) seeds with the bacterial species *Trichoderma asperellum* (T42) and *Pseudomonas fluorescens* (OKC) was found to enhance plant defence responses and to reduce symptom formation on inoculation with powdery mildew (*Erysiphe pisi*) (Patel *et al.*, 2017). In the case of the foliar application of plant growth‐promoting rhizobacteria in pea, the protective effect against powdery mildew correlated with an increase in antifungal compounds (Bahadur *et al.*, 2007).

Taken together, artificially applied microbes can severely affect the extent of powdery mildew colonization in several plant species, either directly via antibiosis or indirectly by the induction of plant defence pathways. Whether pre‐existing indigenous microbial communities in the phyllosphere also do so, thereby possibly limiting the success of powdery mildew pathogenesis, even in interactions with susceptible plant hosts, is currently unknown and deserves thorough investigation.

## Systemic microbial effects on powdery mildew colonization

Some microorganisms can affect the colonization by powdery mildews systemically. This applies to both fungal and bacterial microbes residing in the root/rhizosphere. Particularly well investigated in this respect are arbuscular mycorrhizal fungi and the endophytic growth‐promoting fungus *Serendipita indica* (formerly known as *Piriformospora indica*). In most instances, the presence of these microbes results in fewer powdery mildew symptoms—presumably by inducing a form of ISR in the host plant (Molitor and Kogel, 2009).

Several reports describe the effect that root colonization by arbuscular mycorrhizal fungi has on powdery mildew disease—in part with contrasting outcomes. For example, colonization with the arbuscular mycorrhizal fungus *Funneliformis mosseae* (formerly known as *Glomus mosseae*) was found to protect wheat plants from infection by its powdery mildew pathogen *B. graminis* f. sp. *tritici*. Wheat plants with established mycorrhizal symbiosis showed a reduction in powdery mildew colony formation by 78% (Mustafa *et al.*, 2016, 2017). This effect was associated with a lower number of successful powdery mildew penetration events (resulting in fewer haustoria) and correlated with an enhancement of prototypical plant defence markers (accumulation of polyphenolic compounds and reactive oxygen species at attack sites and up‐regulation of defence marker genes). A similar, but less pronounced, effect (34% protection) was seen with the mycorrhizal fungus *Rhizophagus irregularis* (formerly known as *Glomus intraradices*) in the same set of experiments (Mustafa *et al.*, 2016, 2017). By contrast, in cucumber, the presence of the mycorrhizal symbiont *R. irregularis* had no impact on colony formation of the cucumber powdery mildew pathogen *Podosphaera xanthii* (Larsen and Yohalem, 2004). At the other end of the spectrum, barley plants with an established symbiosis with *Glomus etunicatum* exhibited strongly enhanced susceptibility (formation of asexual conidiospores more than doubled) to the barley powdery mildew pathogen *B. graminis* f. sp. *hordei* (Gernns *et al.*, 2001). Similarly, in standing milkvetch (*Astragalus adsurgens*), pre‐established colonization with *Glomus versiforme* resulted in enhanced susceptibility (disease index, i.e. percentage of leaves covered by pustules) to natural infection by *E. pisi* (Liu *et al.*, 2018). Taken together, arbuscular mycorrhizal fungi appear to enhance or reduce susceptibility/resistance to powdery fungi, possibly as a function of the respective plant species, symbiont species and/or experimental conditions.

More consistent were the results obtained with the endophytic and growth‐promoting fungus *S. indica*. For example, root colonization of barley plants with *S. indica* reduced the extent of powdery mildew infection by *c*. 50%. This effect was correlated with an increase in hypersensitive cell death and unsuccessful penetration events, as well as a decreased number of haustoria (Waller *et al.*, 2005). Subsequently, it was found that *S. indica* systemically primed leaves for alkalinization of the leaf apoplastic space on powdery mildew infection, which might impede powdery mildew pathogenesis by an as yet unknown means (Felle *et al.*, 2009). The presence of *S. indica* in barley roots further led to faster transcriptional responses (activation of defence genes) to powdery mildew attack (Molitor *et al.*, 2011).

In addition to fungi, root‐associated bacteria can stimulate antifungal defence in the phyllosphere. For example, the bacterial strain *Bacillus subtilis* UMAF6639—originally isolated as a phyllosphere endophyte—confers protection to melon plants against cucurbit powdery mildew via the activation of jasmonate‐ and SA‐dependent defence responses (García‐Gutiérrez *et al.*, 2012, 2013). Another example is the α‐proteobacterium *Rhizobium radiobacter*, which is intimately associated with mycorrhizal fungi of the order *Sebacinales*. *Rhizobium radiobacter* conferred enhanced resistance to *B. graminis* (64% decrease in pustules) when root‐inoculated on barley plants (Sharma *et al.*, 2008). Similarly, rhizobacteria of the genus *Streptomyces* (strain AcH 505) have been found to trigger enhanced resistance against powdery mildew (*Microsphaera alphitoides*) attack in pedunculated oak (*Quercus robur*). This was associated with an increase in defence‐associated transcript levels and enzyme (phenylalanine ammonia lyase and peroxidase) activities (Kurth *et al.*, 2014).

## Effects of powdery mildews on root/rhizo‐ and phyllosphere microbiota

In addition to being influenced by the presence of other microorganisms in leaf and root tissues, powdery mildews can also affect microbial communities themselves, possibly via either dedicated or multi‐functional secreted effector proteins (Snelders *et al.*, 2018). Best documented in this respect is the local effect of powdery mildew infection on leaf microbiota. A study with a focus on the phyllosphere of cucumber (*Cucumis sativus*) and Japanese spindle (*Euonymus japonicus*) revealed by culture‐dependent and culture‐independent methods that powdery mildew infection results in larger populations and greater diversity and richness of the leaf‐associated epiphytic bacterial community. Notably, the patterns of these shifts in bacterial community structure were found, in part, to be host plant species specific (Suda *et al.*, 2009). Along similar lines, significant alterations in foliar fungal and bacterial consortia were reported on powdery mildew infection of oak (*Q. robur*) with its powdery mildew pathogen *Erysiphe alphitoides.* This study further revealed a pathobiome network of 13 fungal operational taxonomic units (OTUs) and 13 bacterial OTUs that were interconnected through direct ecological interactions with *E. alphitoides*. At least some of these might be antagonists of *E. alphitoides*, possibly contributing to the protection of oak leaves against powdery mildew attack in natural settings (Jakuschkin *et al.*, 2016). In the pumpkin (*Cucurbita moschata*)–powdery mildew pathosystem, the impact on the richness and diversity of the foliar fungal microbiome was found to correlate with the severity of powdery mildew infection. In weakly powdery mildew‐colonized leaves, an increase in richness and diversity was seen, whereas these parameters decreased in heavily colonized host plants (Zhang *et al.*, 2018). However, data from all of these reports must be considered with caution as they were obtained in case studies from particular natural leaf samples and not from controlled and replicated experiments.

Apart from reports on these local effects, very few studies on the systemic effect of powdery mildew colonization on root‐associated microorganisms exist. So far, these exclusively relate to prominent symbiotic microbes (rhizobia and arbuscular mycorrhizal fungi). Pea plants grown either under laboratory conditions or in the field showed a significant reduction in the degree of spontaneous nodulation by *Rhizobium* spp. This was evidenced by a reduction in the number of nodules per plant, smaller root nodules and lower nitrogenase activity when infected by powdery mildew (*E. pisi*). These effects correlated with the extent of disease severity in both growth regimes (Singh and Mishra, 1992). By contrast, no effect of the powdery mildew (*P. xanthii*) infection of cucumber (*C. sativus*) on the root‐colonizing mycorrhizal symbiont *R. irregularis* was found (Larsen and Yohalem, 2004). It remains to be seen whether the general root and rhizosphere microbial community structure is also affected by powdery mildew infection. In the case of downy mildew (*Hyaloperonospora arabidopsidis*) infection of *Arabidopsis*, the recruitment of three disease resistance‐inducing and growth‐promoting beneficial bacterial species in the rhizosphere has been observed (Berendsen *et al.*, 2018).

## Interactions of powdery mildews with other foliar pathogens

As for the interplay between root and leaf microbiota, comparatively little is known about the interaction of powdery mildew fungi with other (foliar) pathogenic microbes. Arguably most extensively studied in this respect is the interplay of powdery mildews with other powdery mildew fungi, which can lead to the phenomena of ‘induced accessibility’ and ‘induced inaccessibility’ at the cellular level on sequential inoculations. In such experiments, a given powdery mildew species/isolate (the ‘inducer’) is inoculated on a host plant species, followed by a second inoculation with either the same or another species/isolate (the ‘challenger’). In general, host cells that are successfully colonized by a virulent ‘inducer’ species/isolate allow subsequent haustorium formation in the same cell, even by an otherwise avirulent ‘challenger’ species/isolate (‘induced accessibility’). Conversely, cells that successfully defend the primary attack are typically also resistant to a secondary challenge, even with a virulent species/isolate (‘induced inaccessibility’). These phenomena, which are not only restricted to the attacked cell itself, but to some degree extend into neighbouring cells, have been widely studied, using different powdery mildew combinations, tissue types and forms of host resistance (Lyngkjær *et al.*, 2001; Lyngkjær and Carver, 1999a,b; Olesen *et al.*, 2003; Yamaoka *et al.*, 1994). In a similar experimental set‐up as in the sequential powdery mildew inoculation experiments, it was discovered that the colonization of barley leaves by a virulent rust fungus (*Puccinia hordei*) induces enhanced resistance against subsequent powdery mildew infection, exemplified by a reduction in haustorium formation. This effect was found to be local and independent of the virulence status of the powdery mildew challenger isolate (Aghnoum and Niks, 2012).

An interesting example of the competitive interaction of multiple powdery mildew species naturally sharing the same plant hosts is provided by the European oak–powdery mildew pathosystem. Three different, yet closely related, *Erysiphe* species (*E. alphitoides*, *E. quercicola* and *E. hypophylla*) are able to colonize European oak trees (*Quercus* spp.). The three powdery mildew species are of Asian origin, presumably invaded the European continent independently and nowadays competitively colonize the same host spectrum. A multiscale analysis revealed that overall niche separation dominates the interplay of the three powdery mildew species on European oak at the geographic, stand, leaf and within‐leaf level. However, they also partly co‐occur, forming a simple fungal community whose composition and distribution are shaped by host factors, oak developmental stage and environmental conditions (Desprez‐Loustau *et al.*, 2018).

The examples mentioned so far refer to the interplay between two biotrophic pathogens (powdery mildew–powdery mildew or powdery mildew–rust). However, necrotrophic pathogens can also affect the outcome of plant–powdery mildew interactions. For example, established infections with the related necrotrophic fungal pathogens *Septoria nodorum* (the causal agent of Septoria nodorum blotch) and *Zymoseptoria tritici* (the causal agent of Septoria leaf blotch) reduced the severity of subsequent powdery mildew challenges in both field and controlled experiments (Orton and Brown, 2016; Weber *et al.*, 1994). In addition to the interplay with filamentous pathogens, the effect of virus infection on powdery mildew has also been investigated, revealing a complex pattern. Previous infection by *Barley yellow dwarf virus* (BYDV) initially suppressed and then subsequently enhanced the extent of powdery mildew colonization on oat and barley (Potter, 1980). Although the list of examples provided here might not be comprehensive, it is evident that interactions between various diseases is still an underexplored field of research.

## Powdery Mildew Mycoparasites

An obvious case of direct encounter of powdery mildew fungi with other microbes is the presence of microbial species that act as ecto‐ or endoparasites of powdery mildews (mycoparasites) or that otherwise interfere with powdery mildew development. At least 40 fungal species have been reported to antagonize powdery mildew growth, either by antibiosis or mycoparasitism, and some of these are used as biocontrol agents to combat powdery mildew disease (Kiss, 2003). For example, the epiphytic yeast *Pseudozyma aphidis* can act as an ectoparasite of the cucurbit powdery mildew pathogen *P. xanthii*. *P. aphidis* interferes with plant colonization by *P. xanthii* via a transition from yeast‐like to filamentous growth, which results in the formation of long hyphae that parasitize the powdery mildew mycelium (Gafni *et al.*, 2015). *Pseudozyma* spp. act generally through antibiosis, leading to plasmolysis and, consequently, death of powdery mildew cells (Kiss, 2003). However, at least some members appear to pursue a dual virulence strategy by antibiosis and ectoparasitism (Gafni *et al.*, 2015). This also involves the secretion of effector proteins to divert nutrients from the plant host via the powdery mildew pathogen to the fungal hyperparasite (Laur *et al.*, 2018).

The possibly best‐studied powdery mildew mycoparasites are *Ampelomyces* spp. (e.g. *A. quisqualis*), which are widespread pycnidial fungi (Kiss, 1998). Members of this genus attack powdery mildews by invading their hyphae as endoparasites, ultimately leading to a complete necrotizing destruction of the mycelia, thereby preventing completion of the powdery mildew life cycle (Kiss, 2008). The fungus *Paecilomyces farnosus* shows a unique mycoparasite behaviour as it interferes with powdery mildew growth on *in vitro* leaf cultures (under conditions of high humidity), but not on plants in the glasshouse (Szentiványi *et al.*, 2006). Mycoparasites co‐occur with powdery mildews in natural settings, as, for example, reported for the oak powdery mildew pathogen (Topalidou *et al.*, 2016). Such intimate fungal associations may also include species that are seemingly not mycoparasitic (Topalidou *et al.*, 2016). The tritrophic interaction between plant hosts, powdery mildew fungi and their mycoparasites serves as an interesting example of plant and fungal ecology (Parratt *et al.*, 2018; Pintye *et al.*, 2015).

## Conclusions and outlook

A role of associated microbial communities in influencing the outcome of host–pathogen interactions is widely acknowledged, but rarely studied. Although some reports exist regarding the interplay between powdery mildews and other microbes, the global picture is still rather incomplete (Fig. 1). For example, the contribution of the indigenous foliar microbiota to powdery mildew colonization is currently unknown. It is at least conceivable that pre‐existing leaf microbiota limit the infection of adapted powdery mildew species, and possibly even contribute to so‐called non‐host resistance against non‐adapted pathogens. Likewise, the interaction of powdery mildews with other (foliar) pathogens—including other powdery mildew species—is a severely underexplored field of research. With regard to the existing reports, in most instances, antagonistic effects of other microbes on powdery mildews have been found, and there are only a few papers that have described a synergistic effect (Gernns *et al.*, 2001). The majority of published studies have made use of rather poorly defined powdery mildew disease parameters (such as ‘disease index’ and/or ‘infected leaf area’) and lack appropriate quantification of early (entry rate) and late (conidiation) powdery mildew colonization success. In addition, several investigations have been based on single case studies, including a wide variety of different host and pathogen species, often deficient in controlled conditions and/or experimental replication. Thus, there is an urgent need for more systematic approaches, using more sophisticated methods to score powdery mildew infection success, e.g. by quantitative assessment of infection structures (Baum *et al.*, 2011; Seiffert and Schweizer, 2005; Weßling and Panstruga, 2012). Future studies should also involve the model plant *Arabidopsis* for which, to date, no reports exist in this respect. This would allow the addition of a genetic component by testing plant mutants which, for example, do not support ISR or which intercept powdery mildew pathogenesis at a defined stage. Such investigations will probably shift the focus from currently still largely descriptive reports to work that provides more insight at the mechanistic level. A deeper understanding of the mutual interplay between powdery mildew fungi and other microbes holds promise for a better comprehension of fungal ecology and the obligate biotrophic lifestyle, and may also reveal novel possibilities for disease control.
